# Acidic tumor microenvironment and pH-sensing G protein-coupled receptors

**DOI:** 10.3389/fphys.2013.00354

**Published:** 2013-12-05

**Authors:** Calvin R. Justus, Lixue Dong, Li V. Yang

**Affiliations:** ^1^Department of Oncology, Brody School of Medicine, East Carolina UniversityGreenville, NC, USA; ^2^Department of Internal Medicine, Brody School of Medicine, East Carolina UniversityGreenville, NC, USA; ^3^Department of Anatomy and Cell Biology, Brody School of Medicine, East Carolina UniversityGreenville, NC, USA

**Keywords:** cancer, tumor microenvironment, acidosis, proton-sensing G protein-coupled receptors, GPR4, GPR65 (TDAG8), GPR68 (OGR1), GPR132 (G2A)

## Abstract

The tumor microenvironment is acidic due to glycolytic cancer cell metabolism, hypoxia, and deficient blood perfusion. It is proposed that acidosis in the tumor microenvironment is an important stress factor and selection force for cancer cell somatic evolution. Acidic pH has pleiotropic effects on the proliferation, migration, invasion, metastasis, and therapeutic response of cancer cells and the function of immune cells, vascular cells, and other stromal cells. However, the molecular mechanisms by which cancer cells and stromal cells sense and respond to acidic pH in the tumor microenvironment are poorly understood. In this article the role of a family of pH-sensing G protein-coupled receptors (GPCRs) in tumor biology is reviewed. Recent studies show that the pH-sensing GPCRs, including GPR4, GPR65 (TDAG8), GPR68 (OGR1), and GPR132 (G2A), regulate cancer cell metastasis and proliferation, immune cell function, inflammation, and blood vessel formation. Activation of the proton-sensing GPCRs by acidosis transduces multiple downstream G protein signaling pathways. Since GPCRs are major drug targets, small molecule modulators of the pH-sensing GPCRs are being actively developed and evaluated. Research on the pH-sensing GPCRs will continue to provide important insights into the molecular interaction between tumor and its acidic microenvironment and may identify new targets for cancer therapy and chemoprevention.

## Introduction

Solid tumors consist of multiple cell types, such as cancer cells, stromal cells, and immune cells, and a large assortment of extracellular matrix proteins and chemical signals (Hanahan and Weinberg, 2011). The interaction that stromal cells, immune cells, and the extracellular matrix have with cancer cells regulates tumor growth and development. Because of insufficient blood perfusion, hypoxia, inflammation, and glycolytic cell metabolism, the tumor microenvironment has long been known as characteristically acidic (Vaupel et al., 1989; Gatenby and Gillies, 2004; Cairns et al., 2006; Yang et al., 2012). The unique glycolytic metabolism of cancer cells produces an excessive amount of lactic acid (Warburg, 1956). Consequently, the export of protons and lactic acid from tumor cells into the extracellular space by acid-base regulators, such as Na^+^/H^+^ exchangers and monocarboxylate transporters, may lead to acidosis in the tumor microenvironment (Izumi et al., 2003; Boedtkjer et al., 2013). Under normal physiological conditions the pH of blood and tissue is tightly controlled around pH 7.4. However, in diseased tissues such as the tumor microenvironment a local pH range from 5.5 to 7.0 is not uncommon (Vaupel et al., 1989; Gatenby and Gillies, 2004). Moreover, intracellular pH of tumor cells is slightly alkaline in comparison to the extracellular space, which has been demonstrated to facilitate cell proliferation and tumor growth (Tannock and Rotin, 1989; Vaupel et al., 1989; Griffiths, 1991; Griffiths et al., 2001; Webb et al., 2011).

**Figure 1:**
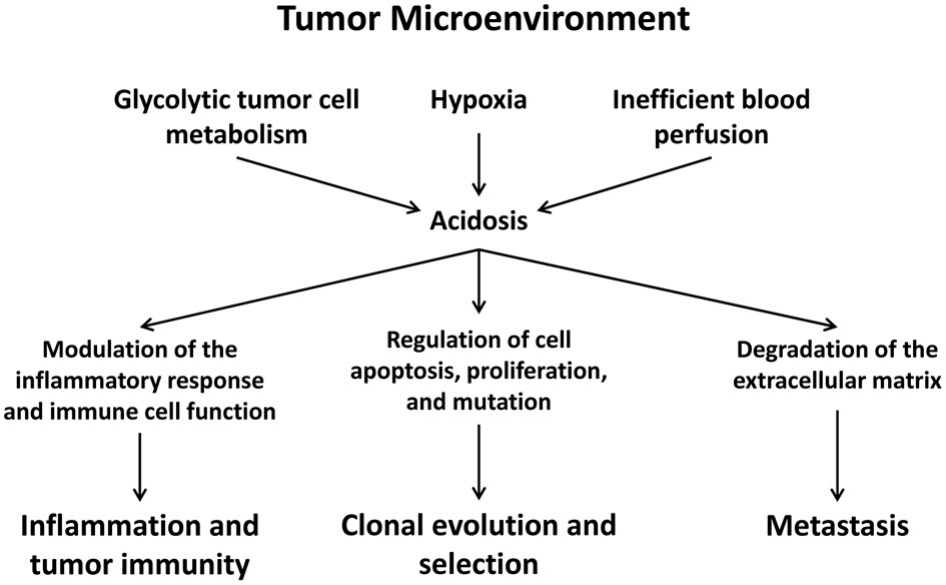
**The acidic tumor microenvironment regulates the proliferation, apoptosis, clonal evolution, and metastasis of cancer cells and modulates inflammation, anti-tumor immunity, and angiogenesis**.

Acidosis in the tumor microenvironment may have many effects on the malignancy and development of a tumor (Figure 1). It has been proposed that exposure to chronic acidosis may facilitate cancer cell clonal evolution by inducing chromosomal instability, clastogenicity, and gene mutations (Morita et al., 1992; Xiao et al., 2003). In addition, acidosis may contribute to metastatic progression by degrading the extracellular matrix (Rozhin et al., 1994; Brisson et al., 2012). On the other hand, acidosis can be cytotoxic, inhibit cancer cell proliferation, and induce stress response and apoptosis (Ohtsubo et al., 1997; Williams et al., 1999; Putney and Barber, 2003; Smallbone et al., 2010). Taken together, acidosis has been described as a defining hallmark of the tumor microenvironment. However, the mechanisms by which cancer cells, immune cells, and blood vessels sense acidosis and respond to it are yet to be completely established but may be advantageous for a more comprehensive understanding of tumor biology.

There are several families of receptors and ion channels that help cells sense extracellular acidosis (Holzer, 2009). In this review we discuss a family of proton-sensing G protein-coupled receptors (GPCRs) including GPR4, GPR65 (TDAG8), GPR68 (OGR1), and GPR132 (G2A). It has been reported that these GPCRs are involved in cancer cell proliferation, apoptosis, metastasis, angiogenesis, immune cell function, and inflammation (Ludwig et al., 2003; Singh et al., 2007; Yang et al., 2007; Mogi et al., 2009; Castellone et al., 2011; Chen et al., 2011; He et al., 2011; Ren and Zhang, 2011; Wyder et al., 2011; Dong et al., 2013).

## Acidosis and tumor cell function

A number of studies have examined the acute and chronic effects of acidosis on tumor cell gene expression, proliferation, apoptosis, and malignancy. In general, cells become stressed in a pH range that deviates moderately from pH 7.4. When the pH range of a tissue significantly falls outside of this category cell function may be affected and apoptosis may be induced. Acute acidosis may inhibit proliferation and increase apoptosis of tumor cells (Ohtsubo et al., 1997; Williams et al., 1999; Putney and Barber, 2003; Smallbone et al., 2010). Chronically, however, acidosis can act as a stressor and select for tumor cells that are resistant to the acidic microenvironment (Moellering et al., 2008; Marino et al., 2012; Wojtkowiak et al., 2012). The mechanism by which tumor cells may overcome acidosis-associated cell death while normal cells perish has been investigated. Autophagy, a catabolic degradation process in which unnecessary organelles and various proteins are recycled, has been described as a mechanism to evade acidosis-related cell death (Marino et al., 2012; Wojtkowiak et al., 2012). Tumor cells may develop chronic autophagy, which allows for acidosis-resistant tumor cells to continue to survive and proliferate (Marino et al., 2012; Wojtkowiak et al., 2012). In addition, several reports connect p53 activity to acidosis-mediated cell death (Williams et al., 1999; Reichert et al., 2002). In tumor cells with p53 mutations acidosis-mediated cell death may be bypassed and cell division may continue to take place, which confers a survival advantage to some tumor cells (Williams et al., 1999; Reichert et al., 2002).

The effects of acidosis on cancer development and progression are complex. They may be separated into acute and chronic exposure related effects as the responses of tumor cells to these conditions are diverse. It has been reported that chronically high lactate in cervical cancers are correlated with a reduction in patient survival (Walenta et al., 2000). In addition, several reports have indicated that chronic systemic buffer therapy may reduce tumor growth and metastasis in SCID or TRAMP mice indicating that chronic acidosis may facilitate tumor growth and metastasis (Ibrahim Hashim et al., 2011; Ibrahim-Hashim et al., 2012). Acidosis has also been demonstrated to increase the secretion of proteases such as cathepsin B in melanoma cells and matrix metalloproteinase-9 (MMP-9) in human osteosarcoma cells (Rozhin et al., 1994; Matsubara et al., 2013), which may facilitate the degradation of extracellular matrix proteins and subsequently accelerate tumor cell invasion. Acidosis has been reported to increase aggressiveness in human melanoma cells. By treating A-07, D-12, and T-22 human melanoma cells with media buffered to pH 6.8, several proteins such as MMP-2, MMP-9, cathepsin B, cathepsin L, VEGF-A, and IL-8 are upregulated and contribute to increased experimental lung metastasis post tail vein injections in athymic nude mice (Rofstad et al., 2006).

On the other hand, acidosis has been reported to induce tumor cell apoptosis, and inhibit cell proliferation and carcinogenesis (Ohtsubo et al., 1997; Zanke et al., 1998; Williams et al., 1999; Putney and Barber, 2003; Smallbone et al., 2010; Matsubara et al., 2013). Episodic and transient acidosis is proposed to be a possible mechanism by which increased physical activity reduces the risk of cancer incidence (Smallbone et al., 2010). It has been reported there is decreased expression of glycolytic genes and increased expression of the tricarboxylic acid (TCA) cycle and electron transport genes in response to acute acidosis in breast epithelial cells (Chen et al., 2008). The gene signature in response to acute acidosis is correlated with a higher survival in breast cancer patients. Furthermore, the response to acute acidosis in prostate cancer cells has been reported to reduce the activation of the oncogene Akt (protein kinase B) (Chen et al., 2008), which is upregulated in a variety of cancers and is important for several processes such as glucose uptake, cell survival, and cell proliferation.

In addition, acidosis in the tumor microenvironment may affect the response of cancer cells to therapy. It has been demonstrated that the efficacy of some chemotherapeutics is affected under acidic pH in comparison to the physiological pH. Acidic extracellular pH may also modulate the charge status of weak acid and weak base chemotherapy drugs and therefore, influence the uptake of these drugs across the cell membrane, in particular, reducing the uptake of weak base drugs and increasing the uptake of weak acid drugs (Reichert et al., 2002; Cairns et al., 2006; Gerweck et al., 2006).

## Acidosis and the inflammatory response

The inflammatory response in the tumor microenvironment has long been known to show both tumor promoting activity as well as anti-tumor activity (Kim et al., 2007; Grivennikov et al., 2010; Hanahan and Weinberg, 2011). How acidosis may affect the activity of immune cells in the tumor microenvironment may be highly valuable in the field of cancer biology and tumor immunology. Inflammatory cells and mediators themselves may further reduce the pH of the tumor microenvironment by increasing glucose uptake and glycolysis as well as stimulating ATP-dependent proton extrusion mechanisms (Grinstein et al., 1991; Imtiyaz and Simon, 2010). Numerous studies indicate that acidosis has multiple effects on the function and response of inflammatory mediators such as neutrophils, monocytes, macrophages, and the complement pathways (Lardner, 2001; Martinez et al., 2006; Jancic et al., 2012).

Acidosis induces the activation of neutrophils through the PI3-kinase/Akt and ERK signaling pathways (Martinez et al., 2006), and it also prompts the release of platelet-activating factor, a potent inflammatory activator, from human neutrophils through the p38 mitogen-activated protein kinase (MAPK) pathway (Owen et al., 2005). Neutrophils themselves may enhance acidosis and hypoxia in the tumor microenvironment by increasing oxygen consumption and subsequently emitting protons following respiratory burst (Van Zwieten et al., 1981; Borregaard et al., 1984). Acidosis may also stimulate the release of IL-1β from monocytes and TNF-α from resident alveolar macrophages both of which are strong inflammatory mediators (Heming et al., 2001; Jancic et al., 2012). Paradoxically, acidosis can also reduce the inflammatory/immune response. In other reports the activity of natural killer (NK) as well as lymphokine-activated killer (LAK) cells is reduced and the release of TNF-α, interferon-γ, IL-10, IL-12 and transforming growth factor-β_1_ (TGF-β_1_) is also diminished by acidosis (Fischer et al., 2000; Muller et al., 2000). Studies also showed that acidosis impairs cytolytic activity and causes anergy of CD8^+^ T lymphocytes (Calcinotto et al., 2012). The mechanisms by which inflammatory/immune cells sense acidosis and respond to it may be of importance in the tumor immunology and inflammation field as this is the environment the immune cells function in.

## Acidosis and blood vessels

Blood vessels formed in the tumor microenvironment are characterized by structural abnormalities, functional defects, and hyperpermeability (Fukumura and Jain, 2007; Nagy et al., 2010). Due to rapid tumor growth and an increased distance between vasculature and cancer cells beyond the oxygen diffusion limit, the tumor microenvironment is hypoxic and acidic (Gatenby and Gillies, 2004). Consequently, hypoxia and acidosis also regulate the generation of new blood vessels from pre-existing blood vessels, a process known as angiogenesis.

Acidosis has been reported to inhibit angiogenesis in the aortic ring vessel outgrowth model (Burbridge et al., 1999). In the presence of vascular endothelial growth factor (VEGF) and basic fibroblast growth factor (bFGF), however, this inhibitory effect may be attenuated and angiogenesis may resume (Burbridge et al., 1999). It has been shown that acidosis increases the expression of VEGF and bFGF in endothelial cells (D'arcangelo et al., 2000). In addition, a separate study found that pancreatic adenocarcinoma cells produce VEGF in response to acidic pH (Shi et al., 2001). The release of VEGF and bFGF in the tumor microenvironment by tumor cells and endothelial cells may compensate for reduced angiogenesis, and increase the rate of blood vessel growth in acidic conditions.

In addition to angiogenesis acidosis also regulates vascular tone. Acidosis-induced coronary arteriolar dilation is mediated by ATP-sensitive potassium channels (Ishizaka et al., 1999). Furthermore, acidosis can stimulate the inflammatory response of vascular endothelial cells in which the pH-sensing GPR4 receptor is involved (Chen et al., 2011; Dong et al., 2013).

## pH-sensing G protein-coupled receptors and tumor cells

**Figure 2:**
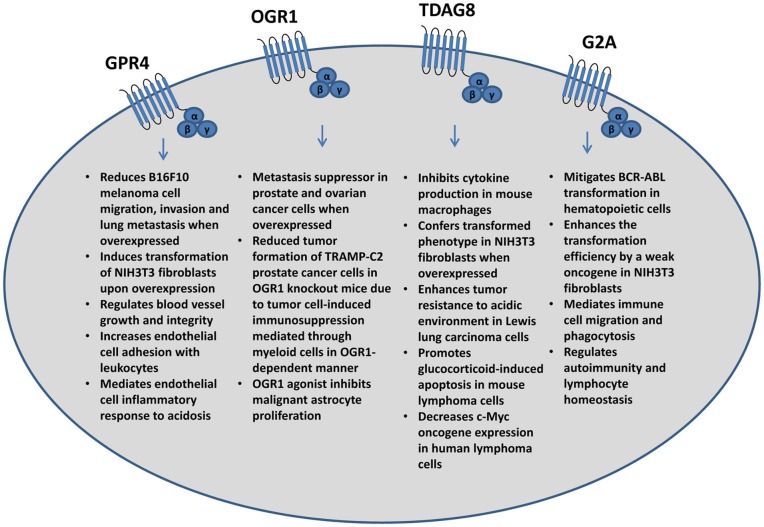
**Major effects of the pH-sensing G protein-coupled receptors on tumor cells and associated stromal cells and immune cells**.

As previously mentioned, the effects of acidosis on tumor cells have been investigated; however, the molecular mechanisms that facilitate these effects are largely unknown due to the complexity of acidosis response. Proton-sensing GPCRs including GPR4, TDAG8 (GPR65), OGR1 (GPR68), and G2A (GPR132) have recently been identified as novel pH sensors that are proposed to be activated by acidic extracellular pH through the protonation of several histidine residues of these receptors (Ludwig et al., 2003; Murakami et al., 2004; Wang et al., 2004; Ishii et al., 2005; Radu et al., 2005; Seuwen et al., 2006; Yang et al., 2007; Liu et al., 2010b; Sun et al., 2010; Saxena et al., 2012). Proton-sensing GPCRs may play a role in tumor development, metastasis, inflammation, and angiogenesis (Figure 2).

GPR4, stimulated by acidosis, has been reported to activate the G_s_, G_12/13_, and G_q_ G-protein pathways (Ludwig et al., 2003; Tobo et al., 2007; Castellone et al., 2011; Chen et al., 2011). When ectopically overexpressed in murine B16F10 melanoma cells, activation of GPR4 by acidosis has several effects on tumor cell function such as decreasing cell membrane protrusions, inhibiting cancer cell migration, and reducing melanoma cell lung metastasis through the G_12/13_/Rho pathway; whereas GPR4 does not significantly inhibit primary B16F10 melanoma growth (Castellone et al., 2011; Zhang et al., 2012). The GPR4 deficiency in mice, however, has been reported to reduce the growth of tumor allografts by impairing angiogenesis from the host mice (Wyder et al., 2011). It has also been shown that ectopic overexpression of GPR4 can transform NIH3T3 fibroblast cells (Sin et al., 2004). Collectively, both tumor-promoting and tumor-suppressing effects of GPR4 have been reported, which may depend on cell type and biological context.

OGR1, another family member, has been reported to suppress metastasis when overexpressed in prostate cancer cells. Following injection into the mouse prostate, PC3 prostate cancer cells that overexpress OGR1 have markedly reduced metastasis to the lung, spleen, and diaphragm (Singh et al., 2007). A separate study found OGR1 expression is lower in distant metastatic lesions in comparison to the primary tumor (Latulippe et al., 2002). Furthermore, overexpression of OGR1 in HEY ovarian cancer cells reduced cell migration and increased cell adhesion to several extracellular matrix proteins (Ren and Zhang, 2011). Similar effects of cancer metastasis suppression by GPR4 and OGR1 may be due to the fact that they share the highest homology between the members of the proton sensing GPCRs.

TDAG8 may also affect tumor development and growth. It has been reported that the overexpression of TDAG8 in Lewis lung carcinoma cells increases tumor growth in mice and may facilitate resistance to acidosis-mediated cell death *in vitro* through protein kinase A (PKA) and ERK related pathways (Ihara et al., 2010). In addition, knockdown of TDAG8 in NCI-H460 human non-small cell lung cancer cells by shRNA decreases cell survival in acidic conditions (Ihara et al., 2010). TDAG8 activation by acidosis also promotes evasion of cell apoptosis under glutamine starvation (Ryder et al., 2012) and its overexpression has been reported to transform immortalized mammary epithelial cells (Sin et al., 2004). Alternatively, TDAG8 expression and activation stimulates glucocorticoid-induced apoptosis (Malone et al., 2004) and inhibits c-Myc oncogene expression (Li et al., 2013) in lymphoma cells and lymphocytes that have high level of endogenous TDAG8 expression. Interestingly, the expression of TDAG8 mRNA is decreased by more than 50% in human lymphoma samples in comparison to non-tumorous lymphoid tissues (Li et al., 2013).

Compared to GPR4, OGR1, and TDAG8, the pH-sensing function of G2A is less defined. Whereas the proton-sensing activity can be detected in G2A-overexpressing cells, the receptor is dispensable for acid sensing in native lymphocytes (Radu et al., 2005). G2A was originally found to affect tumor development and inhibit cell cycle progression at the G2/M stage, leading to G2 accumulation (G2A) and mitosis inhibition (Weng et al., 1998). It is primarily expressed in immune cells and has been known to mitigate BCR/ABL transformation in the mouse leukemia model (Le et al., 2002). Alternatively, some studies indicate that G2A has a transforming ability in NIH3T3 fibroblasts by leading to loss of contact inhibition, anchorage independent growth, survival, and proliferation as well as increased tumorigenicity in mice (Zohn et al., 2000). G2A may also affect the actin cytoskeleton through Gα_13_ and stimulate RhoA dependent actin stress fiber development in swiss 3T3 fibroblasts (Kabarowski et al., 2000). In addition, G2A is mainly studied as an immune regulatory GPCR due to the high expression in lymphoid tissues, which may affect tumor immunology and therefore, possibly affect tumor development and growth.

## pH-sensing G protein-coupled receptors and inflammation

GPR4, OGR1, TDAG8, and G2A have all been reported to regulate inflammatory responses (Mogi et al., 2009; Chen et al., 2011; Onozawa et al., 2011, 2012; Yan et al., 2012; Dong et al., 2013). Recent studies demonstrated that activation of GPR4 by acidosis induced a broad inflammatory response in human vascular endothelial cells as revealed by microarray analysis (Dong et al., 2013). Specifically, GPR4 activation by acidosis upregulates the expression of adhesion molecules, pro-inflammatory cytokines and chemokines, NF-κ B pathway genes, and prostaglandin-endoperoxidase synthase 2 (PTGS2 or COX2) (Chen et al., 2011; Dong et al., 2013). Furthermore, both static cell adhesion assay and flow chamber assay showed that acidosis-induced GPR4 activation leads to the increased endothelial cell adhesion with leukocytes mainly through the G_s_/cAMP/Epac pathway (Chen et al., 2011; Dong et al., 2013).

OGR1 may be involved in tumor immune response. Yan et al. recently demonstrated that OGR1 deficiency in host cells may significantly reduce tumor allograft development of prostate cancer cells in the OGR1 knockout mice (Yan et al., 2012). It was shown in the same study that T cells are required for the rejection of inoculated tumor cells. The authors concluded that OGR1expression in myeloid-derived cells is needed for the immunosuppression induced by prostate cancer cells (Yan et al., 2012).

TDAG8 is highly expressed in immune cells. TDAG8 in mouse peritoneal macrophages has been demonstrated to aid in the process of inhibiting cytokine production from extracellular acidification (Mogi et al., 2009). In addition, TDAG8 deficiency in mice intensifies the type II collagen-induced arthritis and delayed-type hypersensitivity, which demonstrated that TDAG8 may be a negative regulator of the immune response (Onozawa et al., 2011). A putative TDAG8 agonist has recently been identified (Onozawa et al., 2012). Studies showed that the TDAG8 agonist reduces the expression of IL-2 in mouse splenocytes stimulated with anti-CD3 and anti-CD28 antibodies and reduces TNF-α and IL-6 in mouse peritoneal macrophages stimulated with lipopolysaccharide *in vitro* (Onozawa et al., 2012). In a previous study, TDAG8 was reported to play a role in glucocorticoid-induced thymocyte apoptosis in the TDAG8 transgenic mouse model (Tosa et al., 2003). More recently, however, a study demonstrated TDAG8 deficiency does not affect immune system development or glucocorticoid-induced thymocyte apoptosis in the TDAG8 knockout mice (Radu et al., 2006). In eosinophils TDAG8 is the principal proton sensor (Kottyan et al., 2009). Its activity has been demonstrated to increase eosinophil viability and reduce apoptosis which may affect allergic airway disease (Kottyan et al., 2009).

Whereas G2A is also highly expressed in leukocytes, its function in immune cell response to acidosis is unclear. A previous study showed that the pH-sensing capability of G2A is much weaker than other family members (Radu et al., 2006). Instead, a number of reports demonstrated that G2A may mediate the effects of bioactive lipids on immune cells. G2A is required for the chemotaxis of T cells and macrophages toward lysophosphatidylcholine (Radu et al., 2004; Yang et al., 2005; Peter et al., 2008), a pro-inflammatory molecule produced in apoptosis, atherosclerosis and other processes. By increasing macrophage chemotaxis to apoptotic cells G2A may suppress the induction of autoimmunity and inflammation (Peter et al., 2008). G2A was also found to be critical for the lysophosphatidylserine-stimulated clearance of dying neutrophils by macrophages (Frasch et al., 2008). G2A may also function as an anti-autoimmune receptor in mice as G2A-null mice develop a late-onset autoimmune disorder in that G2A may control peripheral lymphocyte homeostasis (Le et al., 2001).

Furthermore, acidosis in inflamed regions may lead to elevated level of pain and increased sensitivity of nociceptors to thermal and mechanical stimuli (Steen et al., 1992; Issberner et al., 1996; Huang et al., 2007; Chen et al., 2009; Hang et al., 2012). The amount of pain may be directly attributed to the amount of acidification. All of the pH sensing GPCRs are expressed in pain relevant loci such as the dorsal root ganglia neurons and particularly small diameter neurons responsible for nociception (Huang et al., 2007). These pH receptors such as TDAG8 and G2A may function to sense high levels of protons for pain sensory and to regulate the development of hyperalgesia (Chen et al., 2009). It was also shown that TDAG8 and its downstream PKA pathway are involved in sensing cancer pain in rats (Hang et al., 2012).

## pH-sensing G protein-coupled receptors and blood vessels

As previously described, angiogenesis in tumors may result in the abnormal development of blood vessels characterized by structural and functional defects (Fukumura and Jain, 2007; Nagy et al., 2010). Furthermore, acidosis may inhibit the growth of blood vessels but the addition of sufficient VEGF and bFGF can rescue the vessel growth (Burbridge et al., 1999). GPR4 has been investigated as one of the contributors to blood vessel growth by regulating the expression of VEGF receptors (Wyder et al., 2011). GPR4 also regulates the integrity and stability of blood vessels. A fraction of GPR4 knockout mouse neonates have increasing rates of spontaneous hemorrhage likely due to the abnormalities of small blood vessels (Yang et al., 2007). Consistently, more fragile and patchy blood vessels were observed in the tumors grown in the GPR4 knockout mice in comparison to that in the wild type mice (Wyder et al., 2011).

Another receptor, OGR1, has been implicated in vascular smooth muscle function. OGR1 has been recently attributed as a major receptor responsible for acidosis stimulation of PGI(2) production and cAMP accumulation in human aortic smooth muscle cells (Tomura et al., 2005). Moreover, OGR1 knockdown with small interfering RNA inhibited acidosis-induced COX-2, PGI(2), and MKP-1 expression in human aortic smooth muscle cells (Liu et al., 2010a). These effects were further found to be regulated by the G_(q/11)_ G-protein signaling pathway (Liu et al., 2010a).

## Therapeutic implications: targeting the acidic tumor microenvironment

As the tumor microenvironment is characteristically acidic, molecular pathways involved in acid-base regulation and pH homeostasis have been explored to devise new approaches for cancer treatment (Izumi et al., 2003; Swietach et al., 2010; Webb et al., 2011). For instance, several groups have recently found that proton pump inhibitors may reduce neoplastic development of esophageal adenocarcinoma and hepatoblastoma (Morimura et al., 2008; Kastelein et al., 2013). In a five-year study a group recognized that proton pump inhibitors such as omeprazole, esomeprazole, rabeprazole, pantoprazole, or lansoprazole lead to a significant reduction in the progression of neoplastic growth and development in patients with Barrett's esophagus (Kastelein et al., 2013). In addition, another group showed that Bafilomycin A1, a vacuolar-type proton pump inhibitor, increases apoptosis in hepatoblastoma cells but not in normal cells, suggesting that it may be a potential treatment to kill cancer cells (Morimura et al., 2008).

Another method that has been tested is basic buffer therapy. In the athymic nude mouse tumor model or TRAMP prostate cancer mouse model, systemic basic buffer therapy was used to increase the pH of the body and tumor microenvironment with the overall goal of reducing tumor development and metastasis (Ibrahim Hashim et al., 2011; Ibrahim-Hashim et al., 2012). Sodium bicarbonate was fed orally over a prolonged period of time, which reduced acidosis in the tumor microenvironment as well as tumor growth, development, and metastasis in the TRAMP mouse model (Ibrahim-Hashim et al., 2012).

Acidosis in the tumor microenvironment has also been exploited to specifically deliver cancer therapeutics and imaging agents to the tumor sites (Vavere et al., 2009; Han et al., 2013; Lin et al., 2013). For example, the use of acid-released nanoparticles, such as polyethylene glycol based hydrogels integrated with imidazole and antagomir, or acid-released nanoparticles carrying gene expression constructs, may increase cancer therapy effectiveness by reducing the systemic effects normally found in chemotherapy (Han et al., 2013; Lin et al., 2013). Recent studies also demonstrate that a type of small peptide, the pH low insertion peptide (pHLIP), can form an α-helix and insert itself into cell membranes specifically at acidic pH. The pHLIP has been used to exclusively deliver imaging agents to the acidic tumor microenvironment for cancer detection in animal models (Vavere et al., 2009).

Since GPCRs are important pharmaceutical targets (Jacoby et al., 2006), modulation of the pH-sensing GPCRs may be highly valuable in the field of cancer therapy and chemoprevention. As discussed in this review, proton-sensing GPCRs play numerous roles in tumor cell behavior, inflammation, and blood vessel growth. Manipulating the proton-sensing GPCRs or their downstream signaling pathways may have anti-cancer therapeutic applicability. Small molecule modulators of the pH-sensing GPCRs are being actively developed and evaluated. Antagonists of GPR4 and agonists of OGR1 and TDAG8 have recently been identified, and these compounds show biological activities that inhibit inflammation and block malignant astrocyte proliferation, among other activities (Taracido et al., 2009; Zhang et al., 2011; Onozawa et al., 2012; Russell et al., 2012; Dong et al., 2013). Moreover, recent progress in nanoparticle delivery of gene expression constructs and oligonucleotides may provide new avenues to target the pH-sensing GPCRs by exploiting the unique acidic tumor microenvironment (Han et al., 2013; Lin et al., 2013). Several reports have found that GPR4 and OGR1 may function as metastasis suppressors (Latulippe et al., 2002; Singh et al., 2007; Castellone et al., 2011; Zhang et al., 2012). Conceivably, acidic pH-released nanoparticles infused with gene constructs to express the pH-sensing GPCRs, such as GPR4 and OGR1, may be specifically delivered into the tumor microenvironment to further minimize systemic toxicity due to the fact that these receptors are only active in acidic environments. Whereas modulators of the proton sensing GPCRs may be of some therapeutic benefit, limitations do exist. For instance, the application of proton sensing G protein-coupled receptor modulators will not address all of the pathogenic issues that may arise from acidosis or altered pH. Acidosis in general is a very complex pathophysiological factor that has a broad effect on various cell processes and functions. Moreover, there are multiple acid-base sensing and transport systems that tightly regulate pH homeostasis at the molecular and cellular level. This makes uncovering specific mechanisms from altered pH and targeting acidosis-related signaling pathways a potentially difficult task. Nonetheless, the implications for this family of proton sensing GPCRs are ever expanding in tumor biology and may prove crucial to understanding how tumor cells, immune cells, and blood vessels sense and respond to acidosis in the tumor microenvironment.

## Author contributions

All authors contributed to data collection and manuscript writing.

### Conflict of interest statement

The authors declare that the research was conducted in the absence of any commercial or financial relationships that could be construed as a potential conflict of interest.
